# Depression in Chronically ill Patients of Railway General Hospital, Pakistan

**DOI:** 10.7759/cureus.11030

**Published:** 2020-10-18

**Authors:** Ashhub H Rana, Osama Babar

**Affiliations:** 1 Psychiatry, Railway General Hospital, Rawalpindi, PAK; 2 Dermatology, Islamic International Medical College (Riphah International University), Rawalpindi, PAK

**Keywords:** depression in chronic illness, management of depression in chronic illness, suicide and depression, age and depression, past psychiatric history and depression, importance of treating depression, depression in pakistan, depression in rawalpindi, depression in ipd, depression in medical disease

## Abstract

According to the World Health Organization (WHO), chronic diseases are the leading cause of death and disability worldwide and estimated to contribute to 73% of all deaths by 2020. In addition to the difficulty in effectively managing chronic diseases, they are often complicated further by the co-morbid depression stemming from the original disease. Depression has the highest burden of disease affecting more than 264 million people worldwide and worsens the burden of co-existing chronic medical diseases as well. A bidirectional relation exists between depression and chronic medical diseases. Statistical mapping of chronically ill patients of Pakistan suggests that 50% of its population suffers from some form of chronic disease. Little data exists for the prevalence of depression in chronically ill patients from most of Pakistan.

Our objectives were to observe the patterns of depression in chronically ill patients and outline the need for intervention (if any) on a population of Railway General Hospital (RGH - a tertiary healthcare hospital in Rawalpindi, Pakistan). We also aimed at finding out the relation (if any) of age, gender, number of hospital admissions, education and effectiveness of medical disease management with depression. A cross sectional study was conducted on patients admitted due to their chronic medical diseases out of a population of 11,000 presenting at the medical OPD of RGH over a period of three months using Patient Health Questionnaire-9 (PHQ -9) Urdu version. About 50% of the patients suffered from moderate to severe forms of depression. A significant positive correlation was found between age and past psychiatric history of illnesses other than depression with depression while no significance was found with number of hospital admissions, gender or education level; 35% had suicidal ideation.

Depression is quite often dismissed, underdiagnosed and leads to a poor quality of life and decrease in cost effectiveness in our population. Pakistan needs to use more resources on managing depression and medical professionals need to change their attitudes in holistically managing the patients. Treating depression is just as important as managing other symptoms of chronic medical diseases.

## Introduction

According to the international classification of diseases (ICD-10), depression is characterized as a mood disorder [1]. Mood is a predisposition characterized by chronic chemical changes in the brain that influence neuronal growth and may be simply referred to as a chronic emotional state [2]. Emotions, however, are acute reactions, states and responses to stimuli that are often influenced by temperamental states [2]. 

While sadness is an emotion, depression is a mood disorder characterized by persistent sadness, low energy, decreased interest in daily activities, decreased pleasure, feelings of guilt and suicidal ideation over a period of at least two weeks, often accompanied by changes in sleep and appetite [1]. According to World Health Organization (WHO), depression affects more than 264 million people of all age groups, and according to the Global Burden of Diseases Report 2010, was considered as the leading cause of burden [3,4]. It has also been shown to significantly increase suicide rate and worsen chronic medical diseases [4]. 

While depression usually evolves in a genetically predisposed individual with the interplay of individual’s psychological and social factors, it has also been linked to the presence of chronic medical diseases [4,5]. People with chronic medical diseases have two to three times the chance of getting a depressive episode when compared to their healthy individuals of their age and gender [4]. Furthermore, it has been shown to have a bidirectional relation with chronic medical diseases where it may not only stem from a chronic medical illness but may cause it as well [5]. 

According to the World Health Organization (WHO), chronic diseases are the leading cause of death and disability worldwide and estimated to contribute to 73% of all deaths by 2020 [6]. Approximately 50% of Pakistan's population is living with a chronic disease [7].

Studies show that depression indents the cost effectiveness and successful management of chronic medical illnesses [5]. It is believed that while chronic medical diseases cause depression due to pathological changes and accompanying poor quality of life, depression may cause chronic medical diseases or worsen it due to associated chemical imbalances. In many cases, it may lead to maladaptive strategies such as substance abuse [5].

## Materials and methods

Research design:

A cross sectional study was conducted from October 2019 to December 2019 in the medical ward of Railway General Hospital (RGH) a teaching tertiary health care hospital in Rawalpindi, Pakistan.

Sample size and Data collection:

Over the 3 month period, out of 11,000 patients presenting to the medical OPD of RGH, the patients that needed admission due to their chronic medical illnesses in the medical ward were chosen. Patients having persistent diseases for more than 3 months were considered while others were excluded [8]. A total of 156 patients were admitted due to chronic diseases, and after exclusion of re-admissions and incomplete forms, a sample of 100 patients was identified.

The sample population was given the Urdu version of the Patient Health Questionnaire (PHQ-9) accompanied by the caring doctors who took individual informed consent and administered the scale.

The PHQ-9 is a standardized screening tool and considers the cardinal symptoms of depression including feelings of sorrow, misery, guilt, lack of pleasure, decrease interest in daily activities, disturbance of sleep and suicidal ideation [9].

Furthermore, the patients were questioned about their education, existing diseases, past psychiatric history, number of admissions in the last year and time spent on the illness and its management.

Ethical Considerations:

All patients were accompanied by their caring doctors and individual informed consent was taken. Patient care was not compromised at any cost and confidentiality was maintained. Furthermore, it was ensured that the patients understood what they were being asked and for what purposes was their data to be used.

Data Analysis:

The responses were analyzed using the IBM Corp. SPSS build 1.0.0.1327.

## Results

Most of the patients presented to the medical OPD in the months of October, 2019 to December, 2019 as a follow up of their routine examinations, medication adjustments and prescription procurement. However a few presented with acute exacerbations of their chronic medical diseases, thus needing admission; they were screened effectively by the PHQ-9 (Urdu Version). The statistical analysis of a hundred patients revealed that 24% were admitted due to uncontrolled diabetes and its complications, 19% due to the exacerbations of chronic obstructive pulmonary disease (COPD), 14% for complications of coronary artery disease (CAD), 8% for the management of chronic liver disease, 9% due to uncontrolled hypertension and debilitating rheumatoid arthritis each, 8% suffering from complications of chronic kidney disease, 5% due to underlying thyroid disease and a 4% for the management of chronic iron deficiency. By using PHQ-9 on the aforementioned patients, we found that the patients with COPD suffered the most at 73.68% (n=14) affected with moderate to severe depression. The depression rates of patients with moderate to severe forms were found as 64.29% (n=9) with coronary artery disease, 62.5% (n=5) in chronic liver disease, 60% (n=9) in diabetes, 50% (n=2) with iron deficiency, 40% (n=14) with underlying thyroid disease and 22.2% (n=2) in rheumatoid arthritis and uncontrolled hypertension each. According to the scale, depression scores of 0-5 interpreted as minimal depression, and 5-9 as mild depression were not considered (cut off value of 10) [9].

Pearson coefficient was used to calculate the relation between depression severity in chronic illness and the number of hospital admissions, past psychiatric history, age, gender, education and marital status (table 1).

**Table 1:** Correlations of Depression using Pearson’s coefficient (2-tailed) **: Correlation is significant at the 0.01 level (two-tailed).

	Age	Gender	Education	Marital Status	Number of hospital admissions	Past Psychiatric History	Depression severity score
Pearson's correlation	0.286**	0.11	-0.055	0.123	0.115	0.288**	1
Significance (two-tailed)	0.004	0.276	0.586	0.222	0.254	0.004	
N	100	100	100	100	100	100	100

A total of 38% patients had no history of previous admissions in the last year while 24% had one, 28% had two, 3% had three and the remaining 7% had four or more admissions due to their poor control and acute exacerbations of chronic medical diseases. A positive correlation was found between previous history of admission and depression but was insignificant (r(98)=0.115, p=0.254; significant at 0.01) (table 1).

A total of 21% patients had a history of psychiatric disorders other than depression. A significant positive correlation was found (r(98)=0.288, p=0.004; significant at 0.01) (table 1).

While 55% (n=31) men suffered minimal to mild forms of depression, 56.82% (n=25) women suffered from moderate to severe forms of depression. A positive correlation of female gender was found with the severity of depression but was insignificant (r(98)=0.110, p=0.276; significant at 0.01) (table 1).

While most of our patients had never received any form of formal education. We found that the level of education negatively correlated to depression but the relation was insignificant (r(98)=-0.55, p=0.586; significant at 0.01) (table 1).

Age, however, significantly correlated with the presence of depression. A positive correlation with age was found (r(98)=0.266, p=0.004; significant at 0.01) (table 1).

Marital status correlated positively but the finding was insignificant (r(98)=0.123, p=0.222; significant at 0.01) (table 1).

**Figure 1:**
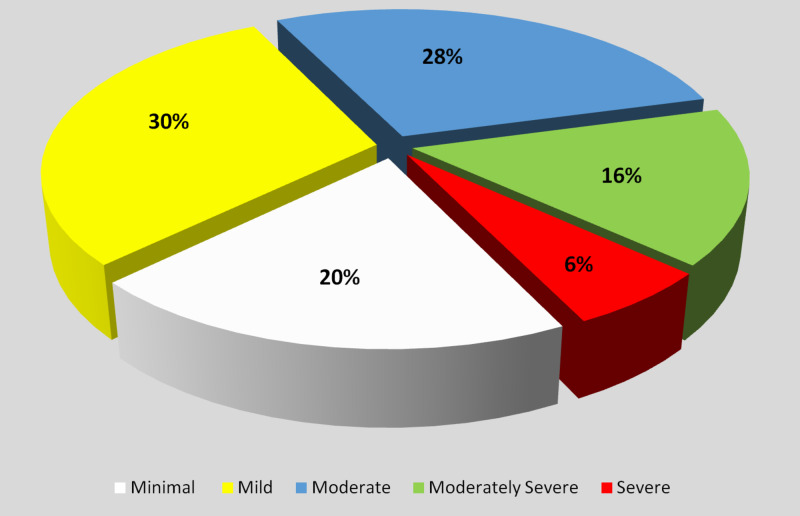
Severity of Depression in chronically ill patients

A general analysis of all the patients revealed that 20% had minimal and 30% mild depression, both of which were not considered depression for this study [9]. While 28% had moderate and 16% moderately severe, 6% suffered from severe forms of depression (Figure 1). An overall prevalence of 50% was calculated in patients suffering from chronic medical diseases regardless of the diseases they had.

As it can be seen in Table 2, when comparing the individual responses, we found that in the last 2 weeks, 57% had decreased interest and pleasure in daily activities with 28% at several days, 12% more than half the days and 17% almost every day. About 57% had low mood and felt depressed with 28% at several days, 12% more than half the days and 17% almost every day. About 73% had sleep disturbances: mainly difficulty falling asleep with 29%, several days, 19% more than half the day, and 25% almost every day. Approximately 84% of the patients complained of having low energy and feeling tired with 30% at several days, 19% for more than half the days and 35% for almost every day. Only 28% had normal appetite with 27% having disturbed appetite on several days, 15% on more than half the days, and 30% almost every day in the last 2 weeks. Out of the 46% that felt like they had failed their families and felt guilty with low self esteem were 25% on several days, 9% on more than half the days, and 12% nearly every day. About 52% said they did not have any difficulty in maintaining attention and focusing on a task with 29% that did on several days, 11% on more than half the days and 8% nearly every day. From the 54% that complained of psychomotor agitation and retardation in the form of fidgeting, restlessness or slowness and quietness, 30% were pointed out on several days while 7% and 17% on more than half the days and nearly every day respectively. Majority denied suicidal ideation at 65% while 19% had suicidal ideation and thoughts of being better off dead than dealing with their illnesses on several days, 9% more than half the days and 7% almost every day in the last two weeks (Table 1).

**Table 2:** Descriptive Analysis of PHQ-9 responses of overall patients

PHQ-9	Not at all	Several days	More than half the days	Nearly every day
Little interest or pleasure in doing things	43%	28%	12%	17%
Feeling down, depressed or hopeless	43%	26%	14%	17%
Trouble falling or staying asleep, or sleeping too much	27%	29%	19%	25%
Feeling tired or having little energy	16%	30%	19%	35%
Poor appetite or overeating	28%	27%	15%	30%
Feeling bad about yourself or that you are a failure or have let yourself or your family down	54%	25%	9%	12%
Trouble concentrating on things such as reading the newspaper or watching television	52%	29%	11%	8%
Moving or speaking so slowly that other people could have noticed. Or the opposite – being so fidgety or restless that you have been moving a lot more than usual	46%	30%	7%	17%
Thoughts that you would be better off dead, or of hurting yourself	65%	19%	9%	7%

An interesting finding to be noted was that even though approximately 90% of the patients initially responded with no suicidal ideation, on insuring them that it was a part of the disease, they reported differently. A reason for this may be found in the cultural and religious taboos associated with having self-harming thoughts [10]. In Pakistan, a very recent change has been made that indicates suicidal attempts are not a crime but a result of mental illnesses needing treatment and not imprisonment. However, it still continues to be a stigma in Pakistan due to its cultural and religious implications [10].

## Discussion

Chronic diseases can be defined as conditions affecting the physical, mental and social well being for a period of at least 3 months [8].

A bidirectional relation exists between chronic medical diseases and depression. However, a unique relation exists for each disease attributing to its pathogenesis and manifestations. Suicidal ideations and suicidal behaviour may manifest from depression attributed to chronic medical diseases [11].

Chronic obstructive pulmonary disease (COPD) is a debilitating chronic disease that is expected by the WHO to become the third most common cause of death by 2030. The disease can have around 80% prevalence of depression depending on the severity [12]. It causes a decrement in the quality of life and cost effectiveness of managing the disease. It is further worsened by the lack of knowledge and management skills for depression among non-psychiatric medical professionals [12]. About 73.68% of our patients with COPD suffered from depression of varying severities and 36.84% reported having suicidal ideation (Figure 3). Depression leads to worsening of COPD as it is seen to affect the activation of diaphragm in patients with respiratory failure [13]. Depression can result from shared risk factors for COPD like genetic predisposition, smoking and environmental stressors or be a direct result of prolonged hypoxemia and oxidative stress to the brain. It may also occur as a result of decreased quality of life majorly due to dyspnea. Alternatively smoking associated with depression may worsen or cause COPD in some patients [14]. Effective management includes use of psychotherapies like Cognitive Behavioural Therapy (CBT), relaxation techniques like progressive muscular relaxation, guided imagery and breathing exercises, physical therapy, group counselling and the use of psychotropic medications along with the management of hypoxemic symptoms of COPD [14]. 

**Figure 2:**
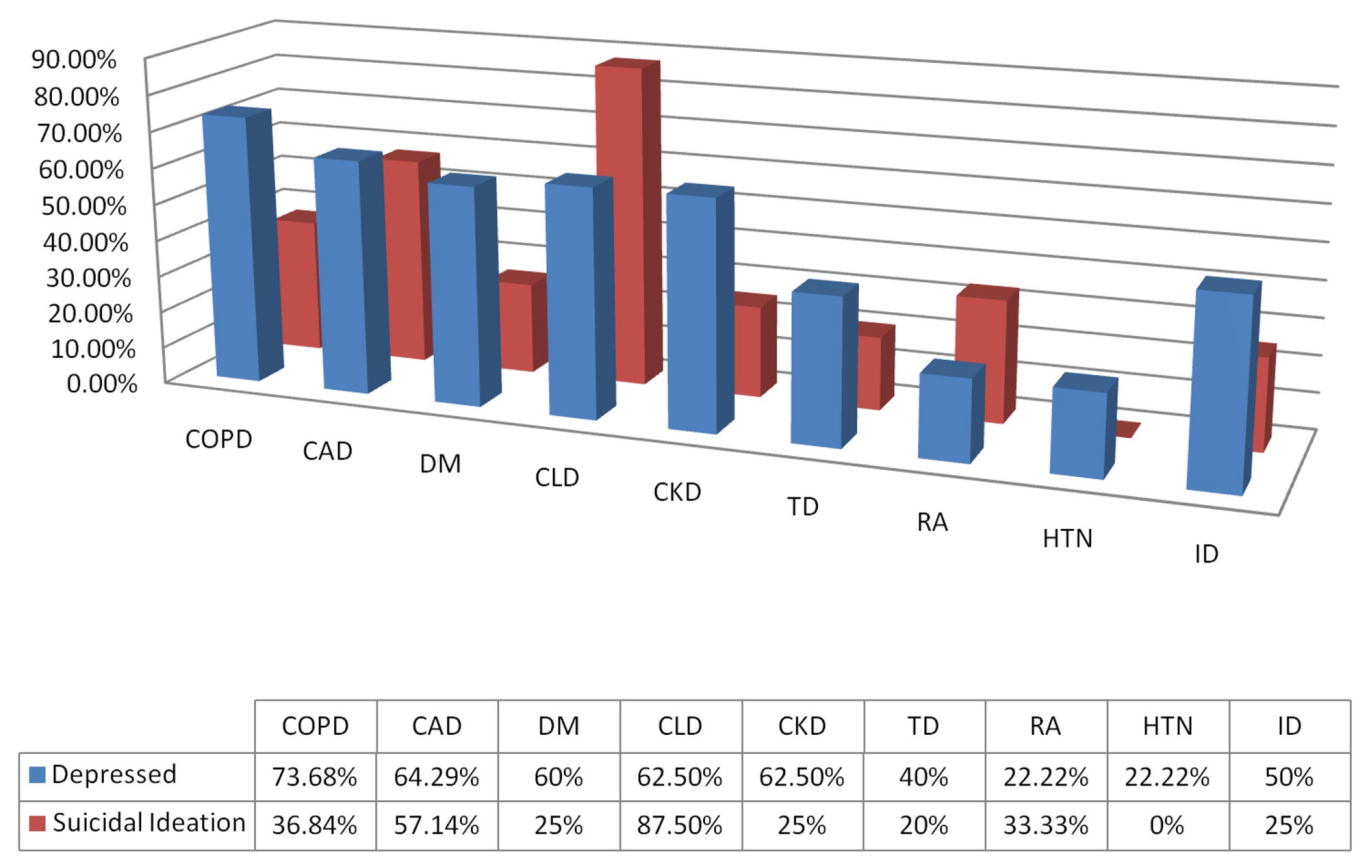
Evaluation of depression and suicidal ideation in chronic medical diseases COPD = Chronic Obstructive Pulmonary Disease; CAD = Coronary Artery Disease; DM = Diabetes Mellitus; CLD = Chronic Liver Disease; CKD = Chronic Kidney Disease; TD = Thyroid Disease; RA = Rheumatoid Arthritis; HTN = Hypertension; ID = Iron Deficiency

**Figure 3:**
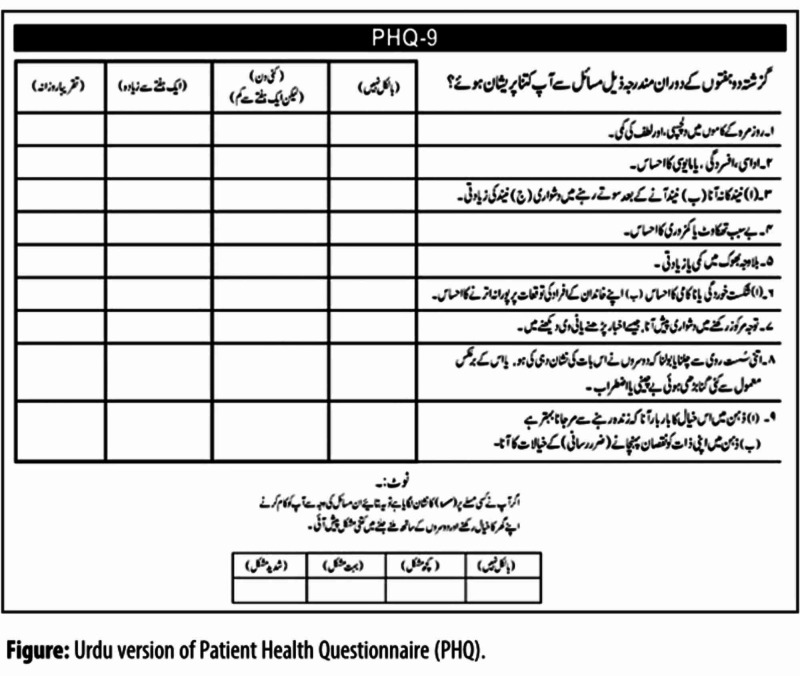
Urdu Version of PHQ-9

Depression is present in around 17 to 44 percent of patients with Coronary Artery Disease (CAD). It has been linked with sudden cardiac failure and considered an individual risk factor for CAD [15]. Persistent depression further worsens the outcomes of the disease. Our study found 64.29% of patients with CAD to be depressed with 57.14% having suicidal ideations (Figure 2). While psychological therapies and drug interventions have shown to significantly improve the quality of life and depressive symptoms, their role in improving survival is still under research [15,16]. Cardiac safe medications should be used when treating depression. 

Patients with long standing diabetes are twice as likely to suffer from a form of depression as healthy individuals [13]. We found that 60% of our patients with diabetes were depressed and 25% reported having suicidal ideation (Figure 2). Poor management of depression and diabetes have shown to influence one another in a bidirectional flow with depression worsening the glycemic control and resulting in associated complications. Shared risk factors like smoking, genetic predisposition, sedentary lifestyle, obesity and lack of a healthy diet have been linked to both of the comorbidities. However, due to negative effect of diabetes on employment and quality of life as a result of complications like diabetic nephropathy, neuropathy, retinopathy and diabetic foot disease, the chances of having depression increase. Psychological therapies like cognitive behavioural therapy CBT and drug therapies like selective serotonin reuptake inhibitors (SSRIs) used to treat depression have shown significant improvement in quality of life as well as the glycemic control with the decrease in the amount and frequency of insulin needed [13]. However, despite the availability of modern techniques of management and early screening tools, only 25% of patients are treated for depression [13].

Chronic liver disease (CLD) includes a number of diseases affecting the liver and eventually resulting in cirrhosis. Patients suffering from CLD are at an increased risk of depression than healthy individuals and it is presumably due to a shared molecular pathway and pathogenesis [16]. Our patients suffered from CLD due to a chronic infection with Hepatitis C Virus (HCV). Approximately 62.5% of them were depressed and 87.5% had suicidal ideation as can be seen in Figure 2. Early management of depression has been linked with better adherence to interferon therapy for HCV infection and improved outcomes of the disease [17].

Patients with chronic kidney disease CKD and end stage renal disease (ESRD) are at greater risks of developing depression. Depression is thrice as common in CKD as healthy individuals [18]. We found 62.5% of our patients with CKD to be depressed while 25% reported having suicidal ideation (Figure 2). It is important to recognize that depression is different in early CKD than ESRD. Management of depression results in improvement of quality of life. However, its efficacy in managing CKD needs more research [18].

Thyroid diseases have been linked to many mental disorders. While the dysfunction of thyroid hormone regulation causes diseases that affect the mental capacity and brain organically through the hypothalamic-pituitary axis and monoamine dysregulation, it has been shown that symptoms of mental disorders like depression persist long afterwards the treatment of the underlying disease. An occurrence of 31 and 56 percent has been observed in hyperthyroidism and hypothyroidism respectively [19]. We found that 40% of our patients with underlying thyroid disease were affected with moderate to severe forms of depression and 20% had suicidal ideation (Figure 2). The use of novel antidepressants like SSRIs and serotonin-norepinephrine reuptake inhibitors has shown good efficacy in treating depression related to thyroid disease [19]. It is important to mention the contraindication of tricyclic antidepressants in hyperthyroid disorders which can worsen the disease. While antidepressants have a major role in treating depression, the underlying thyroid disease should be treated first. Long term psychotherapy sessions (at least 30) have also shown a benefiting response alternatively when indicated [19]. 

Rheumatoid arthritis is a chronic inflammatory disease that affects multiple organ systems and is a cause for lifelong debilitations affecting around 0.3 to 1 percent people depending on the country [20]. Depression in patients is related to activity restriction, debilitating changes of the joints and functional status [13]. A total of 22.22% of our patients with rheumatoid arthritis were depressed and 33.33% thought that they were better off dead than living with their disease (Figure 2). Use of cognitive behaviour therapy with aerobic exercise has shown significant improvement in the quality of life and symptoms. Antidepressants have shown an increasing improvement in the depressive symptoms as well as the symptoms of arthritis [13].

Hypertensive disorders are a great cause of disability worldwide and while they are accelerated with depression, they can be bidirectional in causing depression as well. The biopsychosocial model best explains the causation of depression in hypertensive patients found at 21.3% and 29.8% according to interview based and self reported studies respectively [21]. Shared factors like genetic predisposition, sedentary lifestyle, high fat diet, smoking and inactivity are prominent among both diseases. A total of 22.22% of our hypertensive patients suffered from depression with none of them reporting suicidal ideation (Figure 2). The best management strategy is including psychotherapy like CBT and antidepressant medications as indicated, in conjunction with patient education and vigilant anti-hypertensive therapy [21]. Studies have shown that treating depression improves the adherence to antihypertensive medication and maintains a good blood pressure control, improving the prognosis of the disease [21, 22].

Iron is one of the essential minerals and decreased iron levels have been linked to poor cognitive function and development of mental disorders. The degree of depression directly correlates with the degree of iron deficiency [23]. We found 50% of our patients with iron deficiency severely and 50% mildly depressed (excluded as not having depression), while 25% expressed having suicidal ideation (Figure 2). A rectification of iron deficiency has been linked to improved symptoms with psychotherapy and antidepressant medications warranted many times [24].

Pakistan is a country where mental diseases are stigmatized and in order for people to seek mental help, there needs to be more patient education and general awareness of mental diseases [25]. Furthermore, the attitudes of the treating doctors who mostly dismiss depression in chronic medical diseases and incline towards treating them in preference to depression need to change. Many Pakistani medical practitioners believe supernatural forces influence depression and a great majority believe it to be a normal process of aging and lack of inner strength [26]. Suicidal ideation is highly under-reported due to cultural and religious taboos and is often overlooked [10].

Limitations:

We would like to recognize a few limitations evolving our study. Firstly we used the PHQ-9, which is a self reporting scale and even though we made sure doctors with psychiatric experience monitored it and explained it in context to the chronic diseases, there were no psychiatric evaluations done prior to data evaluation. The patients were however referred for psychiatric evaluation after the survey. Secondly, because of the shared presence of somatic symptoms of depression and most chronic diseases, there might be an overestimation of the results. Lastly, we would have liked to have gone into complete depths of each disease and into the prospects of the best management plans according to our population. We would also have liked to compare the results of our patients with chronic medical diseases with the healthy population.

## Conclusions

Depression is a common mental disorder that affects 50% of our population suffering from a chronic medical disease. The percentage varies for each disease and is directly related to the age of the patients and previous psychiatric history of mental disorders. Suicidal ideation is commonly associated with the illness and worsens the quality of life. Collaborative treatment needs to be reinforced and the rationale that treating depression with the chronic medical diseases benefits general health, aids management of the disease and improves the quality of life, more widely taught and approached. 

Findings of this study were consistent with international literature and frequencies were similar suggesting that depression is also common in our population and the need for integrative care is warranted in Pakistan.
